# Lung function monitoring in the era of respiratory pandemics

**DOI:** 10.1111/cpf.12650

**Published:** 2020-06-29

**Authors:** Lennart K. A. Lundblad, Chung‐Wai Chow

**Affiliations:** ^1^ Meakins‐Christie Laboratories McGill University & THORASYS Thoracic Medical Systems Inc. Montréal QC Canada; ^2^ Division of Respirology Department of Medicine Faculty of Medicine University of Toronto Toronto ON Canada; ^3^ Toronto Lung Transplant Programme Multi‐Organ Transplant Unit University Health Network Toronto ON Canada

1

Oscillometry has been used in adjunct with spirometry in many studies and in clinical practice. Recent events with the spread of a viral respiratory pandemic has caused many clinics to stop using standard spirometry for the fear of aerosol transmission of virus. With recent experiences of our and other groups using oscillometry demonstrating its sensitivity, precision, and ease of use without any forced maneuvers, we suggest that it could be an alternative to spirometry while minimizing exhalation aerosol generation.

Coming on the heels of the SARS, MeRS and H1N1, the respiratory infections caused by the novel SARS‐Cov‐2 virus causing COVID‐19 is remarkable for its scope, speed of infectivity, mortality and geographic spread. It has already overwhelmed the health care capacity of developing and developed countries alike due to the unprecedented need for intensive medical care. Countries have responded with virtual shut down of all but essential services as a means to limit spread of infection. These measures also include postponement of routine follow‐up diagnostic assessments and medical visits for the overall population. Indeed, to free up capacity to care for of the anticipated onslaught of severe COVID‐19, the Toronto Lung Transplant Program made an unprecedented decision on March 16, 2020 to suspend new lung transplants and elected to conduct virtual clinics for all postlung transplant patients with the exception of the few who require in‐person visit and/or essential diagnostic investigations. The measures were taken to minimize risk of infection due to travel to health care facilities as these patients are immunocompromised however, these precautions also mean that routine diagnostic evaluations such as chest imaging, surveillance bronchoscopies and pulmonary function studies are also deferred. Indeed, since March 19, 2020, the pulmonary function laboratories at the Toronto teaching hospitals have been closed until further noticed, which is in accordance with recommendations from expert panels associated with the European Respiratory and the American Thoracic Societies (McCormack & Kaminsky, 2020; McGowan, Sylvester, & Burgos, 2020); While lung transplant patients can self‐monitor pulmonary function with home spirometry (as it is part of the standard postlung transplant care), it is well recognized that home monitoring lacks the quality control of those performed in accredited pulmonary function laboratories. Moreover, in the face of respiratory epidemics and pandemics, how do we care for patients with common chronic lung diseases such as asthma and chronic obstructive lung disease where evaluation by pulmonary function tests is a cornerstone of management?

Oscillometry has emerged as a useful diagnostic tool that has been shown to be highly sensitive to small airway and peripheral lung function (Eddy, Westcott, Maksym, Parraga, & Dandurand, 2019; Foy et al., 2019; Young, Guo, Eddy, Maksym, & Parraga, 2018). Our recent study in lung transplant patients showed that oscillometry outperformed spirometry in detecting physiologic changes associated with biopsy‐proven rejection and improvement following treatment of rejection. In contrast, spirometry was stable or improving in 15 of the 16 episodes of acute rejection (Cho et al., 2020; Usmani, 2020). Oscillometry is very sensitive to alterations in the lung periphery in diseases such as asthma and COPD (Foy et al., 2019; Kuo, Jabbal, & Lipworth, 2019; Lundblad, Miletic, Piitulainen, & Wollmer, 2019; Lundblad, Siddiqui, Bossé, & Dandurand, 2019), a feature that likely could be extrapolated to infections as oscillometry is agnostic to the underlying disease but is specific to the physiologic changes in airway dimensions and lung stiffness, both of which are altered during pulmonary infections.

Have respiratory infections been shown to be detectable by assessing lung mechanics? Indeed, yes! Over 40 years ago a report from Hall et al., 1976 (Hall et al., 1976) showed that pulmonary mechanics measured with oscillometry identified early signs of small airway disease (SAD) in patients infected with H3N2 influenza and that it tracked worsening and improvement of SAD as patients were followed for 5 weeks until resolution when oscillometry normalized in most patients. During the entire period, spirometry did not change significantly (Hall et al., 1976), similar to our transplant study. All patients had an uncomplicated influenza that did not require hospitalization and recovered fully, yet oscillometry was sensitive enough to detect SAD.

In a study of pigs infected with porcine reproductive and respiratory syndrome virus, oscillometry revealed peripheral airway obstruction and reduced lung compliance. The physiology changes correlated with histopathological interstitial pneumonia providing a link between structure and function (Wagner et al., 2011). This link was also illustrated in recent work using magnetic resonance imaging in patients with COPD and asthma where a significant correlation between ventilation defects, oscillometry parameters and quality of life scores were found, providing clinical functional correlations to the structure‐physiologic function link (Eddy et al., 2019; Foy et al., 2019; Young et al., 2018).

The human studies from 1976 were methodologically challenging due to technological limitations necessitating manual recordings on oscilloscopes and analysis with limited computer assistance. Advancements in computerization, signal processing and general scientific progress over the past decades have led to improved technologies with several oscillometers that are now commercially available (Dandurand, Lavoie, Lands, & Hantos, 2019). While normal references values are not as abundantly available as spirometry due to the relative infancy of oscillometry as a diagnostic tool, the growing body of literature suggests that oscillometry offers a highly sensitive assessment of the pathological events such as SAD during lung allograft rejection, asthma, COPD and respiratory infections (Cho et al., 2020; Eddy et al., 2019; Guan et al., 2015; Ochman et al., 2018; Young et al., 2018).

Oscillometry is easy for patients to perform because no effort manoeuvres are required and can thus be repeated more frequently than spirometry. It is generally less time consuming which further reduces exposure time to potential contagions. While quality control is important, operator training is also relatively quick (Wu et al., 2020). A major advantage of oscillometry over spirometry, particularly during respiratory pandemics and epidemics, is that oscillometry is conducted during normal tidal breathing, thus likely significantly reducing generation of aerosols and potential spread of pathogens compared with forced exhalation manoeuvres where the manoeuvre itself and the induced cough contributes to spreading of the contagion (Lindsley et al., 2012, 2016; Yan et al., 2018).

The small airways and peripheral lung are sites of early injury in many respiratory diseases, including viral infections, asthma, COPD, interstitial lung diseases and graft dysfunction. Early detection of SAD will greatly enhance the possibility to treat in a timely manner. The addition of oscillometry to routine pulmonary function monitoring will add further insights to allow us to correlate disease severity with specific measurements in the different oscillometry parameters. In time, we believe that oscillometry will prove to be useful in management of patients with acute and chronic lung diseases while minimizing the public health potential of spreading respiratory infections. Finally, with respect to the current situation, epidemiologists expect the COVID‐19 pandemic to continue in several waves over the next 18–24 months and could affect almost everyone (Giesecke, 2020). We do not believe it is sustainable to maintain optimal patient care without access to pulmonary function testing as objective data is critical for management of patients with chronic lung diseases, for preoperative risk assessment of patients and oncology patients needing life‐saving therapies. Hence, we suggest that use of oscillometry as an alternative to conventional pulmonary function tests could provide high quality information about lung health and lung function while minimize public health risks.

### CONFLICT OF INTEREST

Dr. Lundblad is the clinical Science Director of Thorasys Thoracic Medical Systems, a manufacturer of lung function equipment. Dr. Chow declares no conflicting interests.

REFERENCES

Cho, E.
, 
Wu, J. K. Y.
, 
Birriel, D. C.
, 
Matelski, J.
, 
Nadj, R.
, 
DeHaas, E.
, … 
Chow, C. W.
 (2020). Airway oscillometry detects spirometric‐silent episodes of acute cellular rejection. American Journal of Respiratory and Critical Care Medicine; 201, 1536–1544. 10.1164/rccm.201908-1539oc.32135068

Dandurand, R. J.
, 
Lavoie, J. P.
, 
Lands, L. C.
, & 
Hantos, Z.
 (2019). Comparison of oscillometry devices using active mechanical test loads. ERJ Open Research, 5, 160–2019. 10.1183/23120541.00160-2019
PMC692636431886158

Eddy, R. L.
, 
Westcott, A.
, 
Maksym, G. N.
, 
Parraga, G.
, & 
Dandurand, R. J.
 (2019). Oscillometry and pulmonary magnetic resonance imaging in asthma and COPD. Physiological Reports, 7, 1–11. 10.14814/phy2.13955
PMC632892330632309

Foy, B. H.
, 
Soares, M.
, 
Bordas, R.
, 
Richardson, M.
, 
Bell, A.
, 
Singapuri, A.
, … 
Siddiqui, S.
 (2019). Lung computational models and the role of the small airways in asthma. American Journal of Respiratory and Critical Care Medicine, 200, 982–991. 10.1164/rccm.201812-2322OC
31106566PMC6794099

Giesecke, J.
 (2020). The invisible pandemic. Lancet, 395, e98. 10.1016/S0140-6736(20)31035-7
32539940PMC7200128

Guan, W. J.
, 
Gao, Y. H.
, 
Xu, G.
, 
Lin, Z. Y.
, 
Tang, Y.
, 
Li, H. M.
, … 
Zhong, N. S.
 (2015). Impulse oscillometry in adults with bronchiectasis. Annals of the American Thoracic Society, 12(5), 657–665. 10.1513/AnnalsATS.201406-280OC
25654540

Hall, W. J.
, 
Douglas, R. G.
, 
Hyde, R. W.
, 
Roth, F. K.
, 
Cross, A. S.
, & 
Speers, D. M.
 (1976). Pulmonary mechanics after uncomplicated influenza A infection. American Review of Respiratory Disease, 113, 141–147.124722710.1164/arrd.1976.113.2.141

Kuo, C. R.
, 
Jabbal, S.
, & 
Lipworth, B.
 (2019). I Say IOS You Say AOS: Comparative bias in respiratory impedance measurements. Lung, 197, 473–481. 10.1007/s00408-019-00247-y
31273438PMC6647162

Lindsley, W. G.
, 
Blachere, F. M.
, 
Beezhold, D. H.
, 
Thewlis, R. E.
, 
Noorbakhsh, B.
, 
Othumpangat, S.
, … 
Noti, J. D.
 (2016). Viable influenza A virus in airborne particles expelled during coughs versus exhalations. Influenza and Other Respiratory Viruses, 10(5), 404–413.2699107410.1111/irv.12390PMC4947941

Lindsley, W. G.
, 
Pearce, T. A.
, 
Hudnall, J. B.
, 
Davis, K. A.
, 
Davis, S. M.
, 
Fisher, M. A.
, … 
Beezhold, D. H.
 (2012). Quantity and size distribution of cough‐generated aerosol particles produced by influenza patients during and after illness. Journal of Occupational and Environmental Hygiene, 9, 443–449. 10.1080/15459624.2012.684582
22651099PMC4676262

Lundblad, L. K. A.
, 
Miletic, R.
, 
Piitulainen, E.
, & 
Wollmer, P.
 (2019). Oscillometry in chronic obstructive lung disease: In vitro and in vivo evaluation of the impulse oscillometry and tremoflo devices. Scientific Reports, 9, 11618. 10.1038/s41598-019-48039-x
31406190PMC6690921

Lundblad, L. K. A.
, 
Siddiqui, S.
, 
Bossé, Y.
, & 
Dandurand, R. J.
 (2019). Applications of oscillometry in clinical research and practice. Canadian Journal of Respiratory, Critical Care, and Sleep Medicine, 1–15. 10.1080/24745332.2019.1649607


McCormack, M. C.
, & 
Kaminsky, D. A.
 (2020). Pulmonary function laboratories: Advice regarding COVID‐19. ATS Disease Related Resources, 1. Retrieved from https://www.thoracic.org/professionals/clinical‐resources/disease‐related‐resources/pulmonary‐function‐laboratories.php.

McGowan, A.
, 
Sylvester, K.
, 
Burgos, F.
 et al (2020). ERS 9.1 Statement on lung function during COVID‐19 final with contributors.pdf | powered by box. ERS, 1–5. Retrieved from https://ers.app.box.com/s/zs1uu88wy51monr0ewd990itoz4tsn2h.

Ochman, M.
, 
Wojarski, J.
, 
Wiórek, A.
, 
Slezak, W.
, 
Maruszewski, M.
, 
Karolak, W.
, … 
Zeglen, S.
 (2018). Usefulness of the impulse oscillometry system in graft function monitoring in lung transplant recipients. Transplantation Proceedings, 50(7), 2070–2074. 10.1016/j.transproceed.2017.12.060
30177111

Usmani, O. S.
 (2020). Calling time on spirometry: Unlocking the silent zone in acute rejection post lung transplantation. American Journal of Respiratory and Critical Care Medicine, 201(12), 1468–1470. 10.1164/rccm.202003-0581ed.32209030PMC7301740

Wagner, J.
, 
Kneucker, A.
, 
Liebler‐Tenorio, E.
, 
Fachinger, V.
, 
Glaser, M.
, 
Pesch, S.
, … 
Reinhold, P.
 (2011). Respiratory function and pulmonary lesions in pigs infected with porcine reproductive and respiratory syndrome virus. The Veterinary Journal, 187, 310–319. 10.1016/j.tvjl.2009.12.022
20089425PMC7128265

Wu, J. K.
, 
DeHaas, E.
, 
Nadj, R.
, 
Cheung, A. B.
, 
Dandurand, R. J.
, 
Hantos, Z.
, … 
Chow, C. W.
 (2020). Development of quality assurance and quality control guidelines for respiratory oscillometry in clinic studies. Respiratory Care. 10.4187/respcare.07412.32209708

Yan, J.
, 
Grantham, M.
, 
Pantelic, J.
, 
Bueno de Mesquita, P. J.
, 
Albert, B.
, 
Liu, F.
, … 
Milton, D. K.
 (2018). Infectious virus in exhaled breath of symptomatic seasonal influenza cases from a college community. Proceedings of the National Academy of Sciences, 115, 1081–1086. 10.1073/pnas.1716561115
PMC579836229348203

Young, H. M.
, 
Guo, F.
, 
Eddy, R. L.
, 
Maksym, G.
, & 
Parraga, G.
 (2018). Oscillometry and pulmonary MRI measurements of ventilation heterogeneity in obstructive lung disease: Relationship to quality of life and disease control. Journal of Applied Physiology, 125, 73–85. 10.1152/japplphysiol.01031.2017
29543132
